# Microstructure and Properties of Crack-Free Ti-Modified 6063 Aluminum Alloy TPMS Porous Structures Fabricated by LPBF

**DOI:** 10.3390/ma19091784

**Published:** 2026-04-28

**Authors:** Zian Pan, Yunzhong Liu, Zhenhua Fan, Mingsheng Huang, Wenhao Jiang

**Affiliations:** 1 Guangdong Provincial Key Laboratory for Processing and Forming of Advanced Metallic Materials, South China University of Technology, Guangzhou 510641, China; 18718846552@163.com (Z.P.); zhenhuafan@163.com (Z.F.); hms0829@163.com (M.H.); 13047930629@163.com (W.J.); 2 National Engineering Research Center of Near-Net-Shape Forming for Metallic Materials, South China University of Technology, Guangzhou 510641, China

**Keywords:** laser powder bed fusion, 6063 aluminum alloy, triply periodic minimal surfaces (TPMS), grain refinement, solidification cracking, thermal management

## Abstract

6063 aluminum alloy has broad application prospects in aerospace and microelectronic thermal management systems due to its good thermal conductivity and moderate strength. However, its extremely high hot cracking susceptibility during the laser powder bed fusion (LPBF) process limits the direct manufacturing of complex components. This study proposes a strategy combining material composition modification with advanced structural design. By introducing TiH_2_ nanoparticles (1.0~4.5 wt.%) to modify the 6063 aluminum alloy powder, Diamond-type porous structures based on triply periodic minimal surfaces (TPMS) were successfully fabricated using LPBF technology. The results show that the introduction of TiH_2_ significantly suppresses the solidification cracking of the aluminum alloy. The underlying mechanism is that the L1_2_-structured Al_3_Ti particles, generated by the in situ decomposition of TiH_2_ in the melt pool, provide high-density heterogeneous nucleation sites. This leads to a drastic decrease in the average grain size from 30.46 μm to 0.75 μm (a reduction of 97.5%), achieving a remarkable columnar-to-equiaxed transition (CET). In terms of mechanical properties, the 3.0 wt.% TiH_2_ addition group exhibits excellent plateau stress (28.5 MPa) and energy absorption capacity, which is mainly attributed to the synergistic effect of fine-grain strengthening and Orowan dispersion strengthening. Thermal tests reveal that the thermal conductivity of the 3.0 wt.% group reaches 123 W/(m·K) at 100 °C. The healing of cracks reconstructs the macroscopic heat conduction paths, resulting in a significant improvement in thermal conductivity compared with the unmodified group. This work provides a theoretical reference for the development of high-performance, crack-free, and multi-functional integrated aluminum alloy components via additive manufacturing.

## 1. Introduction

With the dramatic increase in the power density of microelectronic devices [1] and the development of aerospace components towards lightweight and high heat dissipation efficiency [2], the development of multi-functional components that combine excellent mechanical load-bearing capacity, efficient heat transfer performance, and complex geometric features has become a hotspot in advanced materials research [3,4]. Traditional heat dissipation solutions mainly rely on aluminum alloy finned heat sinks or aluminum foam structures fabricated by random foaming processes [5,6]. However, these traditional materials face significant limitations in practical applications. For instance, due to the restricted fluid contact area, the convective heat transfer efficiency of aluminum finned heat sinks is gradually approaching its physical limit [7]. Meanwhile, although aluminum foams possess high porosity, their randomly distributed pore topology makes it difficult to achieve precise parametric control of their mechanical properties [8]. Furthermore, the performance of porous structures highly depends on the microstructural integrity of the matrix material, and any microscopic defects can lead to an unexpected collapse in their mechanical response. These challenges prompt researchers to seek innovative solutions from the dual perspectives of material modification and structural design.

In the field of structural design, triply periodic minimal surfaces (TPMS) structures have exhibited remarkable advantages in enhancing convective heat transfer and energy absorption, owing to their characteristics of zero mean curvature, highly interconnected pore channels, and superior specific surface area [9,10]. For example, Al-Ketan et al. [11] found that sheet-based TPMS structures possess higher load-bearing capacity and more uniform stress distribution compared to traditional strut-based lattices. Samad et al. [12] verified through numerical simulations the significant role of Gyroid-type TPMS structures in enhancing fluid perturbation and improving heat transfer characteristics. In the field of energy absorption, Maskery et al. [13] discovered that TPMS structures display a smoother stress plateau than conventional lattices, effectively avoiding early failure caused by stress concentration. Wang et al. [14] investigated the TPMS structures of TiCN-reinforced AlMgScZr alloys and demonstrated that the incorporation of a ceramic phase significantly enhances the load-bearing capacity of the porous skeleton. Furthermore, the Diamond configuration exhibited superior mechanical performance compared to the Gyroid and Primitive configurations, particularly in dispersing local shear stresses and maintaining stable plateau stress. Although TPMS structures possess significant topological advantages, their complex thin-walled features impose stringent requirements on manufacturing precision and material printability [15].

Laser powder bed fusion (LPBF) is an advanced additive manufacturing technique capable of fabricating complex components, but it inevitably introduces various process-induced defects during fabrication, which have become a key bottleneck restricting the performance improvement of LPBF-fabricated parts. As summarized in previous works [16,17], these common defects include process-induced porosity (such as lack-of-fusion voids, gas pores and keyhole pores), hot cracking, strong crystallographic texture, coarse columnar grain growth, as well as residual stress and part distortion. The root causes of these defects are closely related to the unique non-equilibrium solidification characteristics of LPBF: the extremely high thermal gradient, rapid solidification shrinkage, solute segregation during rapid cooling, as well as inappropriate process parameters and feedstock impurities, which lead to the formation of these detrimental defects and significantly deteriorate the mechanical performance of the final products.

In the field of material design, manufacturing high-performance aluminum alloy components using laser powder bed fusion (LPBF) technology faces severe metallurgical challenges. 6XXX series aluminum alloys (e.g., 6061, 6063) are considered ideal materials for heat dissipation components due to their good balance between strength and thermal conductivity [18]. However, Li et al. [19] pointed out that 6063 aluminum alloy has a wide solidification temperature range, making it highly susceptible to forming penetrating solidification cracks under the extremely high cooling rates and steep temperature gradients of LPBF. Hyer et al. [20] further clarified that the solute segregation of alloying elements leads to the formation of weak liquid films at grain boundaries, which induces hot tearing under residual tensile stress. To overcome this hot cracking susceptibility, Uddin et al. [21] attempted to use high-temperature substrate preheating technology to reduce thermal gradients, but this method often weakens the subsequent age-hardening effect of the material.

In recent years, achieving grain refinement through the introduction of heterogeneous nucleants has been proven to be an effective approach to suppress cracks in aluminum alloys. Qbau et al. [22] successfully achieved crack-free forming of commercial 6061 aluminum alloy by adding 0.15 wt.% Sc. Pan et al. [23] indicated that Ti and its derivatives, as highly efficient refiners, can induce a significant columnar-to-equiaxed transition (CET). The inoculation strategy proposed by Mair et al. [24], which modifies the powder surface with nucleants, significantly improved the crack resistance of high-strength aluminum alloys. Furthermore, the introduction of nanoparticles can not only suppress cracks but also enhance matrix performance through dispersion strengthening. For instance, Liu et al. [25] significantly improved material strength by dispersing LaB_6_ nanoparticles in a copper matrix; Sun et al. [26] achieved a synergistic enhancement of strength and ductility in an aluminum alloy using TiB_2_ particles.

Although there have been studies on the densification of Ti-modified aluminum alloys, systematic research on the microstructural evolution of TiH_2_-modified 6063 aluminum alloy within complex TPMS porous structures and its impact on the coupled “mechanical-thermal” performance remains relatively scarce. In particular, the action mechanism of hydrogen gas generated by the decomposition of TiH_2_ on melt pool stability [27], as well as the contribution of refined grains to the load-bearing efficiency of the thin-walled struts in Diamond structures [28], still require in-depth exploration. Therefore, in this work, TiH_2_ was chosen as the modifier because it has unique advantages: compared with the effective but expensive Sc/Zr-based refiners and the easily agglomerated TiB_2_ nanoparticles added externally, TiH_2_ is cost-effective and can in situ form well-dispersed Al_3_Ti nuclei, which are very suitable for our research on the economic and efficient modification of the alloy. In addition, TiH_2_ generates hydrogen pores during the decomposition process, which is a characteristic that distinguishes it from other commercial modifiers, so it is very necessary to study it. In addition, how to maximize the retention of the high thermal conductivity of 6063 aluminum alloy while eliminating cracks is a crucial step in realizing the engineering application of this material.

Addressing the aforementioned challenges, this study proposes a synergistic material-structure strategy. At the material level, modified powders with different TiH_2_ doping levels (1.0~4.5 wt.%) were prepared via low-energy ball milling, and their effects on microstructure, crystallographic texture, and crack suppression were systematically investigated. At the structural level, the Diamond-type TPMS configuration was selected to evaluate its energy absorption behavior under quasi-static compression and heat dissipation performance under steady-state conditions. Compared to bending-dominated topologies (such as the Primitive structure), the stretching-dominated Diamond lattice provides a more uniform stress distribution under compressive loads. This characteristic minimizes structurally induced localized failures, thereby offering a reliable benchmark for evaluating the intrinsic strengthening mechanisms brought by the TiH_2_ modification. In addition, its complex thin-walled features exhibit a high hot-cracking susceptibility during the laser powder bed fusion (LPBF) process, making it an ideal geometry for assessing the crack-suppression efficacy of the modified alloy. This work aims to reveal the interaction between the fine-grain strengthening mechanism induced by TiH_2_ and the inherent properties of TPMS structures, providing a design basis for the development of high-performance, lightweight heat dissipation components.

## 2. Materials and Methods

### 2.1. Powder Preparation

**Figure 1:**
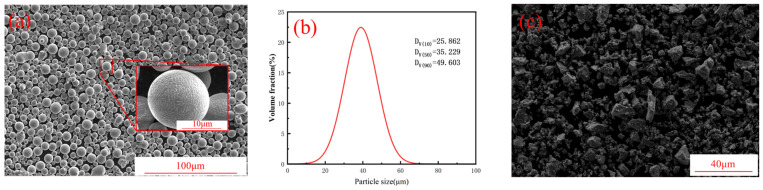
Characterization of the raw powders: SEM micrographs of (**a**) AA6063 and (**c**) TiH_2_; (**b**) particle size distribution of AA6063.

The powder feedstock used for laser powder bed fusion (LPBF) in this study was TiH_2_/AA6063 composite powder. The 6063 aluminum alloy (AA6063) powder produced via gas atomization was supplied by CNPC POWDER Co., Ltd., Shanghai, China. Its chemical composition is listed in Table 1. The scanning electron microscopy (SEM) image in Figure 1a reveals that the powder exhibits a typical spherical morphology with a smooth surface. The particle size distribution measured by a laser particle size analyzer (HORIBA LA960S, manufactured by HORIBA, Ltd., Kyoto, Japan) is presented in Figure 1b, showing a size range of 19.9~59.0 μm with an average particle size of 35.23 μm. The moderate particle size and high sphericity indicate that the AA6063 powder is highly suitable for the LPBF process.

**Figure 2:**
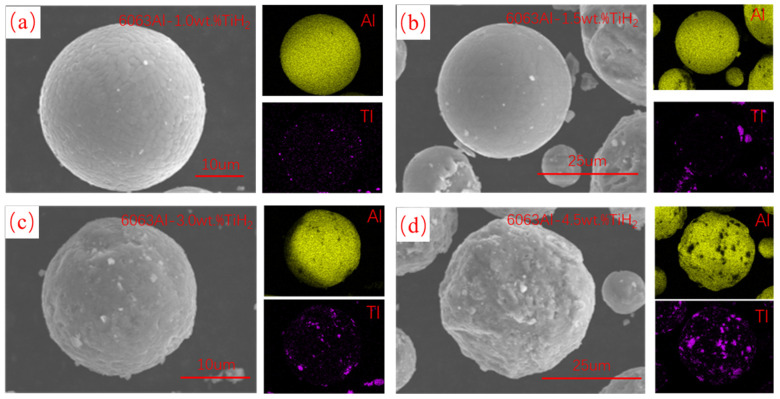
SEM images of the TiH_2_/AA6063 composite powders with different TiH_2_ mass fractions: (**a**) 1.0 wt.%, (**b**) 1.5 wt.%, (**c**) 3.0 wt.%, (**d**) 4.5 wt.%.

**Figure 3:**
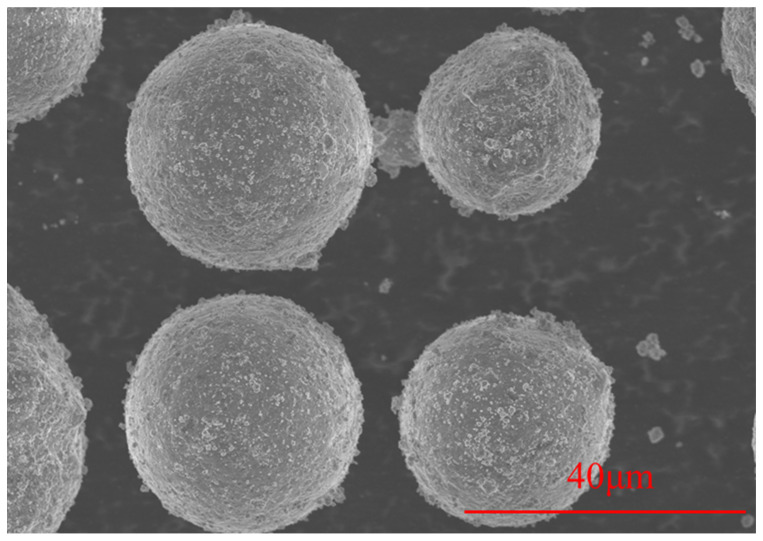
SEM micrographs of the TiH_2_/AA6063 composite powder with a mass fraction of 4.5 wt.%.

In this study, TiH_2_ particles were introduced to modify the AA6063 powder, aiming to improve its printability while enhancing the compressive mechanical properties and heat dissipation performance of the fabricated material. The TiH_2_ powder has an average particle size of 500 nm and a purity of ≥99.9%. The composite powders were prepared via a low-energy ball milling process. Powders with different mixing ratios were placed into a planetary ball mill (QM-3SP4, manufactured by Nanjing Chishun Science & Technology Development Co., Ltd., Nanjing, China). The milling process was conducted at a rotation speed of 180 r/min, with a ball-to-powder weight ratio of 5:1 for 5 h. Four groups of TiH_2_/AA6063 composite powders were prepared with different TiH_2_ mass fractions: 1.0 wt.%, 1.5 wt.%, 3.0 wt.%, and 4.5 wt.%. The SEM images in Figure 2 indicate that the attachment density of TiH_2_ particles on the AA6063 powder surface increases alongside the addition level. Furthermore, Figure 3 shows the morphology of the 4.5 wt.% TiH_2_ composite powder processed under the specified ball milling parameters, revealing a uniform particle distribution that verifies the effectiveness of the mixing process.

For brevity, the as-built samples fabricated from the composite powders with 0, 1.0, 1.5, 3.0, and 4.5 wt.% TiH_2_ additions are hereafter denoted as the 0 wt.%, 1.0 wt.%, 1.5 wt.%, 3.0 wt.%, and 4.5 wt.% samples, respectively.

### 2.2. Modeling of the TPMS Porous Structures

The TPMS structural models used in this study were constructed using MATLAB R2022a software (MathWorks, USA), and the Diamond-type structure was selected from the common TPMS lattice models. The mathematical expression of the Diamond-type TPMS lattice surface is given by [29]:

*φ*d(*x*, *y*, *z*) = cos(*X*)cos(*Y*)cos(*Z*) − sin(*X*)sin(*Y*)sin(*Z*) = *c* (1)

where *X* = *α*·2π·*x*, *Y* = *β*·2π·*y*, and *Z* = *γ*·2π·*z*. The parameters *α*, *β*, and *γ* control the size and shape of the TPMS surface, and (*x*, *y*, *z*) represents the spatial coordinates. The parameter *c* determines the thickness of the thin-walled lattice structure. When *φ* (*x*, *y*, *z*) = *c* = 0, the surface corresponds to the exact triply periodic minimal surface. For the condition of −*c* ≤ *φ* (*x*, *y*, *z*) ≤ *c* (*c* ≠ 0), the surface undergoes an offset of *c* units, and the enclosed volume constitutes the TPMS unit cell. By repeating the single unit cell along the *x*, *y*, and *z* axes, a multi-cell TPMS lattice structure can be generated. The Diamond-type porous structure model constructed in this study has overall dimensions of 15 × 15 × 15 mm^3^ and consists of 2 × 2 × 2 unit cells, with each unit cell measuring 7.5 × 7.5 × 7.5 mm^3^. The volume fraction (VF) is fixed at 30%. The calculation method is as follows:

(2) $$VF=\frac{V_{lattice}}{V_{solid}}$$

where *V_lattice_* is the spatial volume enclosed by the two isosurfaces, and *V_solid_* is the volume of the corresponding cubic domain [30].

**Figure 4:**
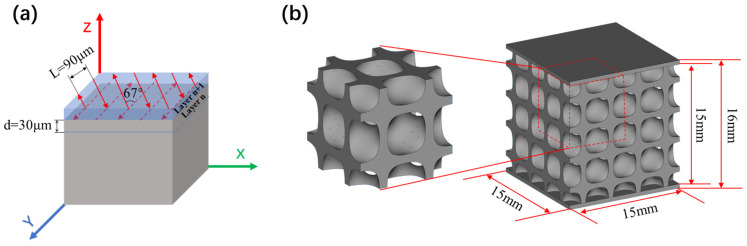
LPBF manufacturing strategy and sample design: (**a**) laser scanning strategy; (**b**) designed Diamond structure model.

The constructed structural model is shown in Figure 4b. In addition, a dense plate with dimensions of 15 mm × 15 mm × 0.5 mm was added to both the top and bottom surfaces of the porous structure model. This design aims to preserve the integrity of the porous structure when the samples are cut from the substrate and to facilitate subsequent metallographic observations.

The dimensions of the constructed Diamond structure were measured using Materialise Magics 24.0 software, and the measurement results are listed in Table 2. The results indicate that the actual volume fraction of the model is slightly lower than the preset value of 30%, which might be attributed to the variations in measurement accuracy among different software. However, since the numerical difference is minor, it can be considered negligible during the sample manufacturing process.

### 2.3. Laser Powder Bed Fusion Process

The samples were fabricated using an EOS M290 LPBF system (EOS GmbH Electro Optical Systems, Munich, Germany), and its main technical parameters are listed in Table 3. As illustrated in Figure 4a, the laser scanning path was rotated by 67° between consecutive layers, and the substrate preheating temperature was set to 180 °C [31].

**Figure 5:**
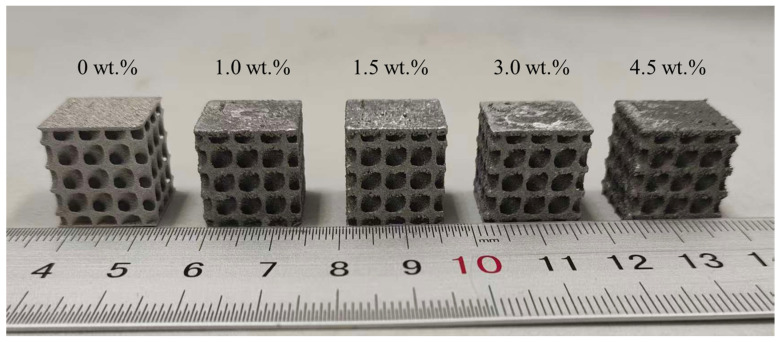
Photograph of the fabricated lattice structure sample.

The fabricated samples are shown in Figure 5. All samples maintained their structural integrity with no visible surface cracks, indicating that the processing parameters listed in Table 3 are suitable for printing this TPMS structural model.

### 2.4. Material Characterization

To remove unmelted residual powders within the porous structures, all samples were treated with an ultrasonic cleaning process. The samples were immersed in deionized water and cleaned for 15 min while maintaining the water temperature at approximately 27 °C [32].

**Figure 6:**
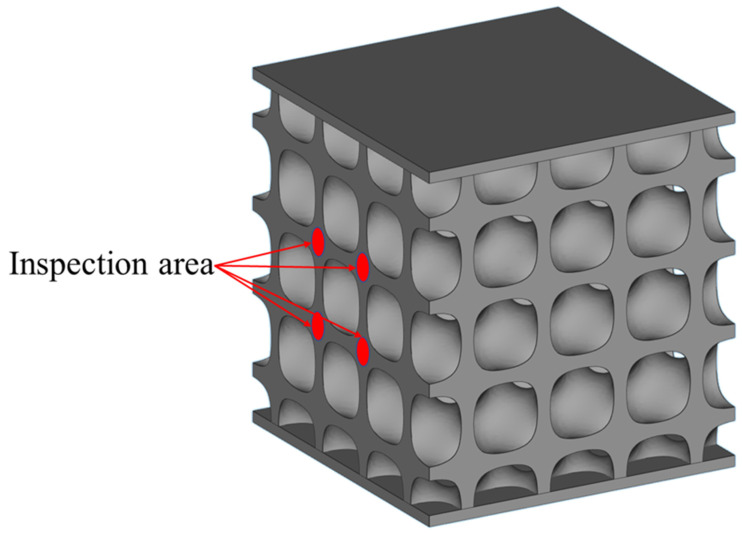
Surface roughness measurement of the samples.

Following the cleaning process, the length and width of the samples with different compositions were measured using a vernier caliper. The height of the samples was not recorded due to significant measurement errors caused by the dimensional changes during the wire electrical discharge machining (WEDM) process used to remove the samples from the substrate. The surface roughness of the samples was measured using a 3D surface profiler (RTEC Up S-Dual, RTEC Instruments, San Jose, CA, USA), as shown in Figure 6. To ensure the reliability of the measurement results, four representative regions on the lattice nodes of each sample were selected as the measurement areas.

Subsequently, the sample surfaces were ground with abrasive papers and then mechanically polished using an alumina suspension. An optical microscope (OM, Leica DM 15000M; Leica Microsystems GmbH, Wetzlar, Germany) was utilized to examine the distribution of cracks and pores on the sample surfaces.

To detect internal pores and lack-of-fusion defects within the porous samples, non-destructive testing was performed using an X-ray micro-computed tomography (Micro-CT) system (nanoVoxel 5000, Sanying Precision Instruments Co., Ltd., Tianjin, China) [33]. Prior to scanning, the samples were fixed on the sample stage to ensure no displacement occurred during the scanning process. The scanning parameters were set as follows: a tube voltage of 80 kV, a tube current of 100 μA, an exposure time of 1.4 s, a sample rotation angle of 360°, and a rotation step size of 0.2°. The projection images were reconstructed using the equipment’s built-in software to obtain 3D volume data. After reconstruction, image analysis software (VGSTUDIO MAX 3.4) was employed to segment and quantitatively analyze the defects, statistically evaluating their equivalent diameters and spatial distributions. To ensure statistical reliability, at least one representative region was scanned for each sample.

The samples used for electron backscatter diffraction (EBSD) required further electropolishing after mechanical polishing [34]. The electrolyte was prepared with methanol and perchloric acid at a volume ratio of 4:1, and the electropolishing was conducted at 15 V and 243 K. The grain orientation and average grain size were calculated using Aztec Crystal 2.1 software. The accelerating voltage was set to 20 kV. The scanning step size was 1.75 μm for the coarse columnar grains and 0.06 μm for the fine short-rod or equiaxed grains.

### 2.5. Thermal Performance Testing

**Figure 7:**
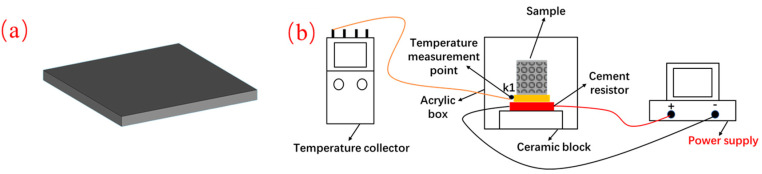
(**a**) Sample geometry for the LFA thermal diffusivity measurement, and (**b**) schematic diagram of the heat dissipation experimental setup.

To meet the dimensional requirements of the testing equipment, the top surfaces of the as-built samples were cut using wire electrical discharge machining (WEDM) to obtain dense plates with dimensions of 10 × 10 × 0.5 mm, as shown in Figure 7a. The thermal diffusivity of each sample plate was measured at 30 °C, 80 °C, and 100 °C using a laser flash apparatus (LFA 467, NETZSCH, Selb, Germany). The specific heat capacity at the corresponding temperatures was determined using a differential scanning calorimeter (DSC 214 Polymer, NETZSCH, Selb, Germany). Finally, the thermal conductivity of the samples was calculated using the following equation [35]:

(3) $$\lambda =\alpha \times \rho \times C_{p}$$

where *λ* is the thermal conductivity, *α* is the thermal diffusivity, *ρ* is the density, and *C_p_* is the specific heat capacity.

The heat dissipation experimental setup is illustrated in Figure 7b. A power supply with a constant power output of 4.0 W was used to continuously heat a cement resistor (10 Ω). A copper plate with dimensions of 20 × 20 × 1 mm was placed on top of the cement resistor, and the sample was positioned on the upper surface of the copper plate. To minimize uneven heat transfer, thermal grease (Thermalright, Taipei, Taiwan, China, thermal conductivity of 13.8 W/(m·K)) was applied between all contact surfaces during each experiment. To prevent heat conduction from the bottom of the cement resistor, a ceramic block was placed underneath it to act as a thermal insulation layer. The sample was enclosed in an acrylic box to ensure a completely sealed environment. The copper plate was primarily heated by thermal conduction from the underlying cement resistor; the heat was then transferred to the sample via conduction, and the sample subsequently dissipated the heat through thermal radiation and natural convection. A K-type thermocouple was attached to the side of the copper plate to monitor the temperature changes, and the data were recorded using a UT325 multi-channel temperature logger (UNI-T, Dongguan, China). The experiments were conducted inside a building located in Guangzhou, Guangdong Province, China (23°9′35″ N, 113°20′17″ E).

### 2.6. Mechanical Properties Testing

The samples with different compositions were subjected to compressive testing using an AG-IC 50 kN testing machine (Shimadzu Corp., Kyoto, Japan). The compression rate was set at 0.45 mm/min, and the gauge length was 15 mm. To eliminate the influence of the building direction, the compressive load was applied along the building direction (*z*-axis) for all samples. Three replicate tests were conducted for each group to obtain the compressive stress–strain curves. Since the actual cross-sectional area of the TPMS structure varies continuously with the cross-sectional position, it is difficult to determine experimentally [36]. Therefore, the nominal cross-sectional area of a fully dense block (i.e., 15 mm × 15 mm) was uniformly adopted for the stress calculations. Although this approach yields “apparent” stresses rather than the true localized stresses within the internal struts—which are actually substantially higher than the calculated values—it remains a standard methodology for assessing the bulk engineering performance and energy absorption capacity of cellular materials. Given that all experimental groups in this study share identical macroscopic dimensions and Diamond topology, using the nominal area provides a consistent baseline, ensuring that the comparisons of mechanical performance across different TiH_2_ modification levels are scientifically valid.

## 3. Results and Discussion

### 3.1. Surface Morphology and Defect Analysis

#### 3.1.1. Macroscopic Dimensions and Surface Roughness

**Figure 8:**
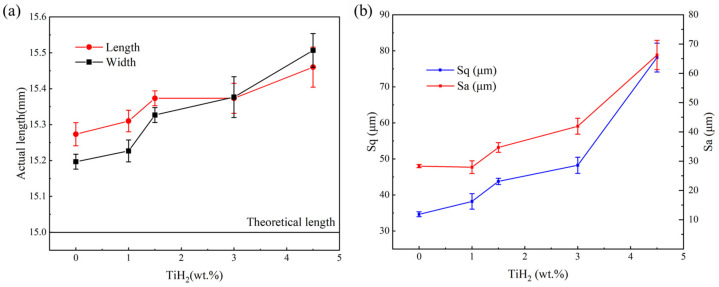
Effect of initial TiH_2_ mass fraction on (**a**) sample dimensions, and (**b**) areal surface roughness (*Sa*) and root-mean-square roughness (*Sq*).

The dimensions of the different samples measured using a vernier caliper are shown in Figure 8a. The unmodified 0 wt.% sample exhibited relatively good dimensional fidelity, with average measured length and width of 15.27 mm and 15.20 mm, respectively (closely approaching the theoretical design dimension of 15.0 mm). Meanwhile, as illustrated in Figure 8b, the surface roughness of this sample group was at a relatively low level within this study, with an areal surface roughness (*Sa*) of approximately 28 µm and a root-mean-square roughness (*Sq*) of about 34.5 µm. However, with the gradual increase in the TiH_2_ mass fraction in the composite powders, the actual as-built dimensions of the samples began to show a significant positive deviation. At an addition level of 4.5 wt.%, the sample dimensions reached their peak expansion, with the length and width measuring 15.46 mm and 15.51 mm, respectively. Concurrently, Figure 8b indicates that the surface roughness exhibited a strictly monotonic increasing trend with the TiH_2_ addition. The *Sa* and *Sq* values of the 4.5 wt.% group increased substantially compared to those of the 3.0 wt.% group, reaching 56 µm and 66 µm, respectively.

**Figure 9:**
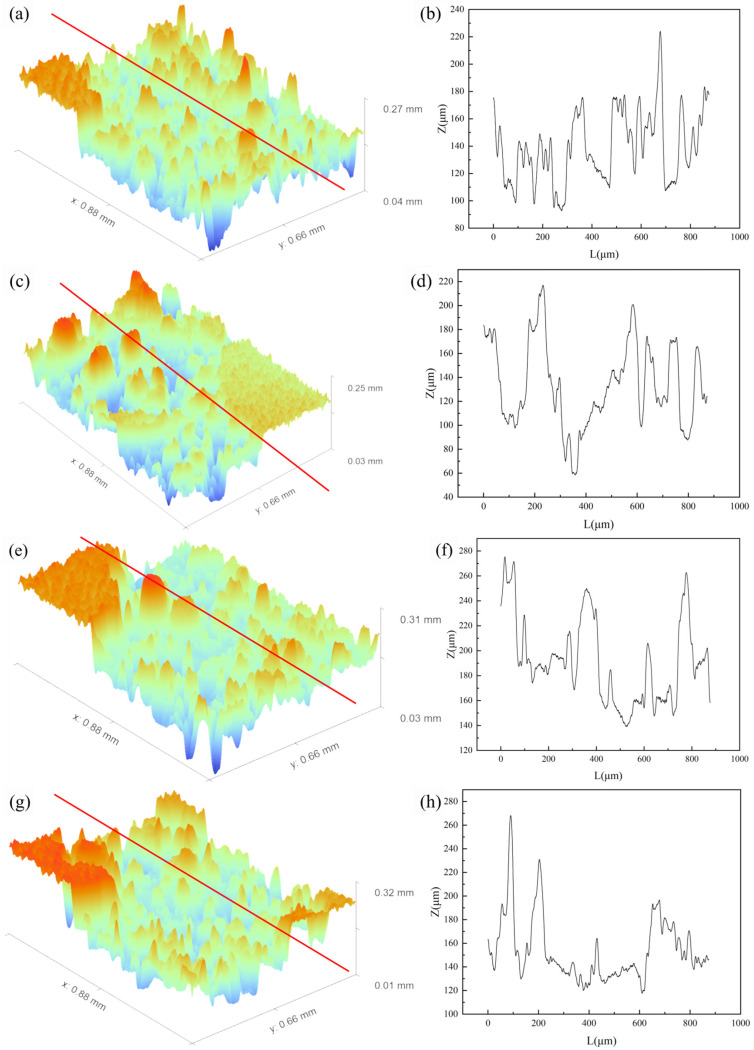

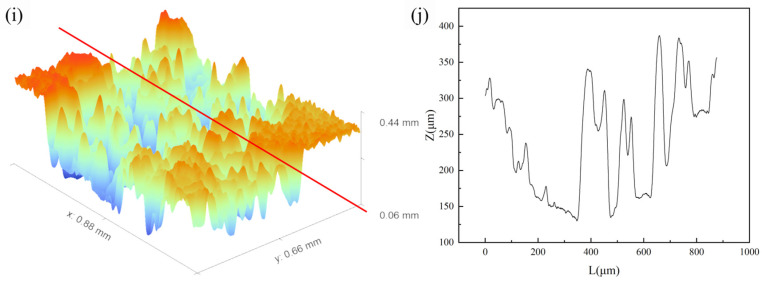
3D surface morphologies and corresponding linear roughness profiles of the samples with various TiH_2_ mass fractions: (**a**,**b**) 0 wt.%; (**c**,**d**) 1.0 wt.%; (**e**,**f**) 1.5 wt.%; (**g**,**h**) 3.0 wt.%; (**i**,**j**) 4.5 wt.%. The red lines in the 3D morphologies indicate the extraction paths for the roughness profiles.

To further analyze this surface quality deterioration behavior, Figure 9 displays the 3D surface profiles of the samples and their corresponding horizontal cross-sectional height extraction curves. The results intuitively corroborate the evolution trend of the roughness data: the surface fluctuations of the unmodified samples were relatively gentle with good continuity; whereas, with the increase in TiH_2_ concentration, the peak-to-valley drops (absolute height difference along the *z*-axis) on the surface were significantly amplified, presenting much more severe peak-like micro-geometric fluctuations. This synchronous phenomenon of abnormal macroscopic dimensional expansion and microscopic roughness deterioration is primarily attributed to the intense thermal decomposition and dehydrogenation reaction induced by the TiH_2_ additive under extremely high-energy-density laser irradiation [37]. During the extremely short laser dwell time, a massive amount of high-temperature hydrogen gas was released in situ. As this gas escaped outward, it not only generated a recoil pressure but also triggered intense gas perturbations within the narrow molten pool and on its free surface. This outgassing perturbation, coupled with the Marangoni convection driven by the temperature gradient between the center and the edge of the molten pool [38], significantly reduced the surface tension stability of the molten pool’s liquid surface and exacerbated the spattering behavior of the liquid metal. Because high-kinetic-energy spatters and surrounding incompletely melted powder particles were heavily captured by the rapidly advancing solidification front and adhered to the surfaces of the as-built structures, it ultimately led to the deviation in macroscopic dimensions and the significant increase in surface roughness.

#### 3.1.2. Metallographic Observation and Quantitative Porosity Analysis

**Figure 10:**
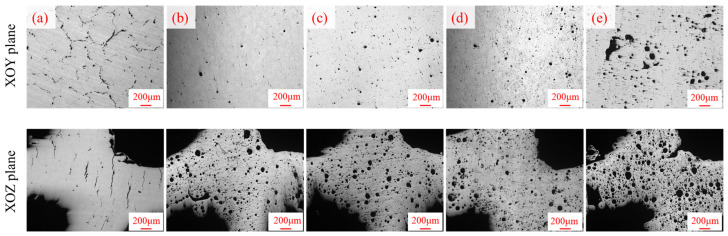
Optical micrographs of the Ti-containing 6063 aluminum alloy with different TiH_2_ mass fractions: (**a**) 0 wt.%, (**b**) 1.0 wt.%, (**c**) 1.5 wt.%, (**d**) 3.0 wt.%, and (**e**) 4.5 wt.%.

Metallographic observations were conducted on the samples with different TiH_2_ mass fractions (0 wt.%, 1.0 wt.%, 1.5 wt.%, 3.0 wt.%, and 4.5 wt.%). The optical microstructures on the XOY plane (perpendicular to the building direction) and the XOZ plane (parallel to the building direction) are shown in Figure 10.

As observed in Figure 10, the unmodified 0 wt.% sample exhibited extremely poor as-built quality. Its XOY plane was covered with irregular network cracks, while the XOZ plane showed longitudinal solidification cracks penetrating multiple melt layers. The crack density was quantitatively analyzed by gray threshold segmentation using ImageJ 1.8.0. The crack densities on the XOY plane and the XOZ plane were obtained as 2.8% and 1.6% respectively. This is primarily attributed to the wide solidification temperature range of the 6063 aluminum alloy. Under the extremely high cooling rates and temperature gradients of the LPBF process, the molten pool is highly prone to forming coarse epitaxial columnar grains. The grain boundaries are highly susceptible to liquid film tearing at the final stages of solidification, resulting in an extremely high hot cracking susceptibility [39]; however, typical spherical gas pores were sparsely distributed in this group. In contrast, when an appropriate amount of TiH_2_ (e.g., 1.0 wt.%, 1.5 wt.%, and 3.0 wt.%) was introduced, the elongated macroscopic and microscopic cracks in the micrographs were completely eliminated, replaced by fine circular pores dispersedly distributed within the matrix. This defect mode transition from “crack-dominated” to “pore-dominated” demonstrates the effectiveness of the modifier in suppressing hot tearing. Nevertheless, when the TiH_2_ addition reached 4.5 wt.%, the intense hydrogen evolution reaction led to molten pool instability, resulting in the appearance of large-sized agglomerated cavities and irregular interconnected pores in the microstructures. These large defects can cause severe stress concentration and significantly reduce the effective heat transfer cross-sectional area of the material, subsequently leading to an overall degradation of the as-built quality.

**Figure 11:**
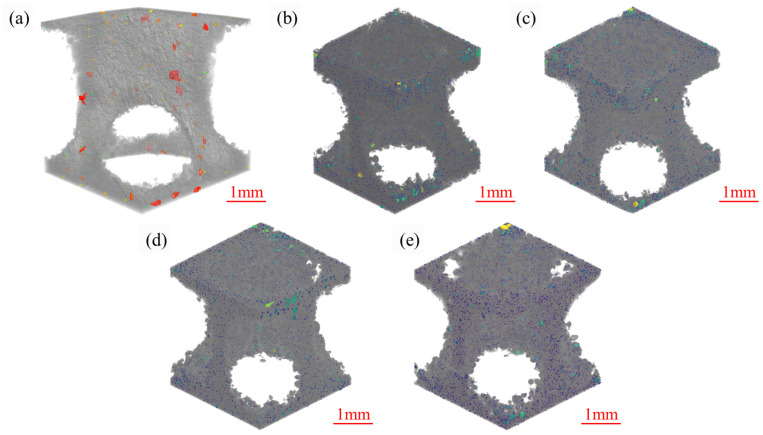
Three-dimensional (3D) XCT reconstructed images showing the porosity distribution in the samples with different initial TiH_2_ mass fractions: (**a**) 0 wt.%, (**b**) 1.0 wt.%, (**c**) 1.5 wt.%, (**d**) 3.0 wt.%, and (**e**) 4.5 wt.%.

Figure 11 displays the porosity distribution of the samples with different TiH_2_ additions. The number of pores in the 0 wt.% group was much lower than that in the other groups; however, it contained some irregularly shaped and large-sized voids, some of which even exceeded 200 μm in diameter. With the introduction of TiH_2_, although the total number of voids increased, they transitioned from large-volume, irregularly shaped printing defect cavities (i.e., keyhole pores and lack-of-fusion defects) to fine, nearly spherical hydrogen-induced gas pores.

**Figure 12:**
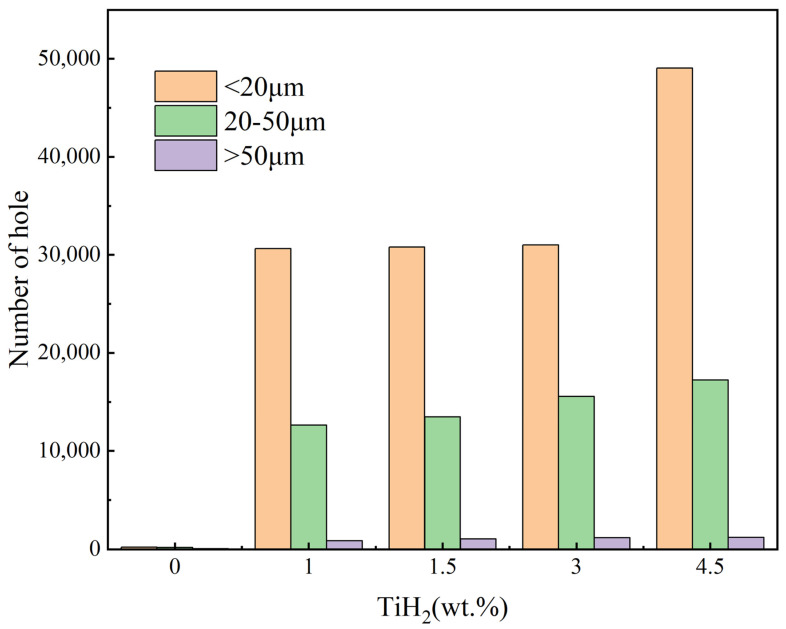
XCT pore size distribution histograms of the samples with different initial TiH_2_ mass fractions.

The XCT pore size distribution histograms presented in Figure 12 quantify the dynamic evolution of hydrogen-induced gas pores generated during the LPBF process. For the 1.0 wt.%, 1.5 wt.%, and 3.0 wt.% groups, the internal pore size distributions were highly consistent, predominantly consisting of fine hydrogen-induced gas pores with equivalent diameters of <20 µm (with a stable frequency of approximately 30,000). Meanwhile, the number of medium-sized pores (20–50 µm) remained at about 15,000, and large-sized pores (>50 µm) were relatively scarce. These statistical results indicate that within this optimal addition range, a steady-state kinetic balance was achieved between the in situ precipitation rate of hydrogen gas and its buoyancy-driven escape rate under the rapid cooling conditions. However, when the TiH_2_ addition increased to 4.5 wt.%, this defect equilibrium state was disrupted. As shown in Figure 12, the frequency of pores <20 µm in the 4.5 wt.% group exhibited a nonlinear, sharp surge, with the total number approaching 50,000. From a physical mechanism perspective, this confirms that the intense thermal decomposition of excessive TiH_2_ released a massive amount of hydrogen gas, far exceeding the solid solubility limit in the molten aluminum. Under the extremely high cooling rates of the LPBF process, the diffusion time for gas molecules within the liquid phase was severely restricted. Consequently, numerous hydrogen gas bubbles failed to float upward and escape from the molten pool’s free surface in time. Instead, they were extensively captured by the rapidly advancing solid–liquid solidification front, thereby forming a high-density hydrogen-induced pore network within the matrix. Therefore, it is imperative to strike an optimal balance in the addition amount between suppressing destructive macroscopic cracks and controlling the micro-porosity caused by dehydrogenation by-products, thereby optimizing the comprehensive performance of the as-built components.

### 3.2. Microstructural Evolution

#### 3.2.1. Phase Composition and Grain Refinement Mechanism

**Figure 13:**
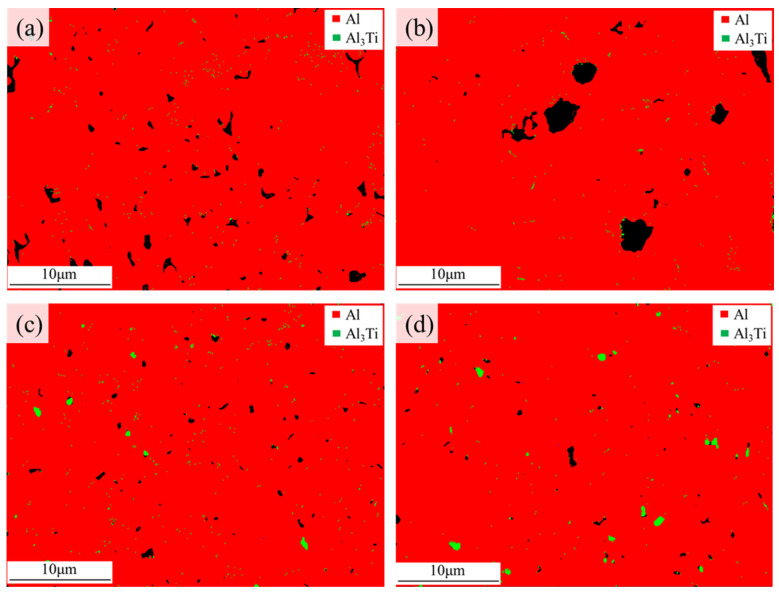
EBSD phase distribution maps on the XOZ plane of the samples with different initial TiH_2_ mass fractions: (**a**) 1.0 wt.%, (**b**) 1.5 wt.%, (**c**) 3.0 wt.%, (**d**) 4.5 wt.%. The black areas represent pore defects.

Comprehensive crystallographic characterizations of the samples were conducted using electron backscatter diffraction (EBSD). Figure 13 illustrates the EBSD phase distribution maps of the samples with four different initial TiH_2_ mass fractions. At the 1.0 wt.% and 1.5 wt.% additions, the Al_3_Ti particles were fine and exhibited a relatively dispersed distribution within the matrix. When the TiH_2_ addition increased to 3.0 wt.%, the density of the Al_3_Ti phase increased significantly, indicating that more Ti elements participated in the in situ reaction. Nevertheless, these particles still maintained a well-dispersed characteristic within the matrix without obvious large-scale agglomeration. According to the edge-to-edge matching (E2EM) model in crystallography, the in situ synthesized L1_2_-Al_3_Ti compound and the face-centered cubic (FCC) α-Al matrix possess an extremely low lattice mismatch and favorable crystallographic orientation relationships (ORs). Their typical crystallographic ORs are generally expressed as {111}Al // {112}Al_3_Ti, <110>Al // <110>Al_3_Ti, and {111}Al // {112}Al_3_Ti, <110>Al // <021>Al_3_Ti [40]. Such highly consistent, coherent or semi-coherent ORs enable them to act as highly effective heterogeneous nucleation sites [41].

**Figure 14:**
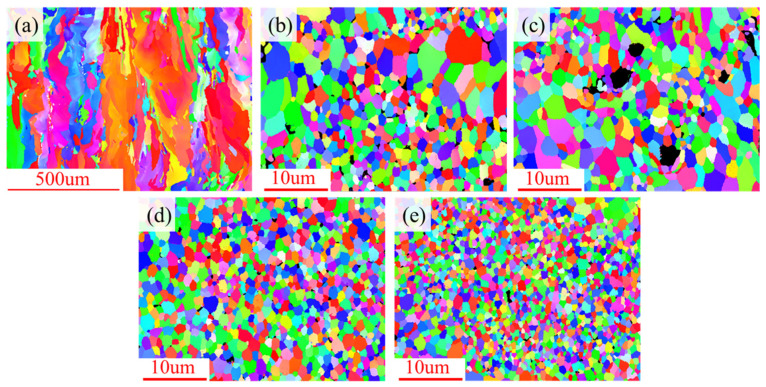
EBSD inverse pole figure (IPF) maps on the vertical cross-sections of the samples with different initial TiH_2_ mass fractions: (**a**) 0 wt.%, (**b**) 1.0 wt.%, (**c**) 1.5 wt.%, (**d**) 3.0 wt.%, and (**e**) 4.5 wt.%.

**Figure 15:**
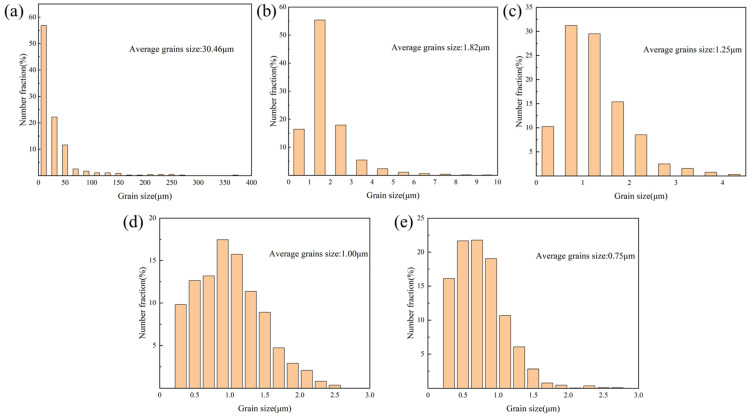
Grain size distributions of the samples with different initial TiH_2_ mass fractions: (**a**) 0 wt.%, (**b**) 1.0 wt.%, (**c**) 1.5 wt.%, (**d**) 3.0 wt.%, and (**e**) 4.5 wt.%.

Driven by these high-density nucleation sites, the solidification behavior of the alloy underwent a fundamental transition. Figure 14 and Figure 15 present the EBSD inverse pole figure (IPF) maps and grain size distribution charts on the vertical cross-sections of the samples, respectively. The results indicate that under the steep temperature gradient of the molten pool during the LPBF process, the unmodified 0 wt.% AA6063 exhibited coarse columnar grains growing epitaxially along the building direction, with a large average grain size of 30.46 µm. Upon the introduction of TiH_2_, the critical thermodynamic energy barrier required for nucleation was significantly reduced due to the presence of abundant Al_3_Ti particles within the molten pool, leading to a distinct alteration in grain morphology. The average grain sizes of the 1.0 wt.%, 1.5 wt.%, 3.0 wt.%, and 4.5 wt.% groups were refined to 1.82 µm, 1.25 µm, 1.0 µm, and 0.75 µm, respectively, representing a maximum reduction of 97.5%. This achieved a significant columnar-to-equiaxed transition (CET) within the molten pool [42]. Additionally, the combined effect of Marangoni convection further suppressed preferential grain growth along the heat flow direction [43].

**Figure 16:**
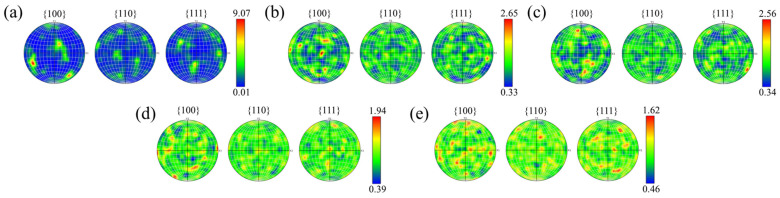
EBSD pole figures (PFs) on the vertical cross-sections of the samples with different initial TiH_2_ mass fractions: (**a**) 0 wt.%, (**b**) 1.0 wt.%, (**c**) 1.5 wt.%, (**d**) 3.0 wt.%, and (**e**) 4.5 wt.%.

Meanwhile, the quantified results from the pole figures (PFs) in Figure 16 reveal that the 0 wt.% group exhibited a strong {001} texture, with a texture intensity of 9.07. In contrast, the texture intensity of the modified groups decreased progressively with TiH_2_ addition, dropping to a minimum of 1.62. This suggests that the original epitaxial growth trend has been effectively suppressed, and the matrix transforms into a fine-grained network with random orientation, thereby reducing the anisotropy of the samples [44]. However, Figure 13 also indicates that when the TiH_2_ addition was increased to 4.5 wt.%, the Al_3_Ti particles underwent coarsening and grew in size due to the constraints imposed by the solid solubility limit and diffusion kinetics of Ti in the aluminum matrix. Such large-sized brittle phase clusters not only weakened the nucleation potency but also tended to act as microcrack initiation sites during subsequent mechanical loading.

#### 3.2.2. Grain Boundary Characteristics and Residual Stress

**Figure 17:**
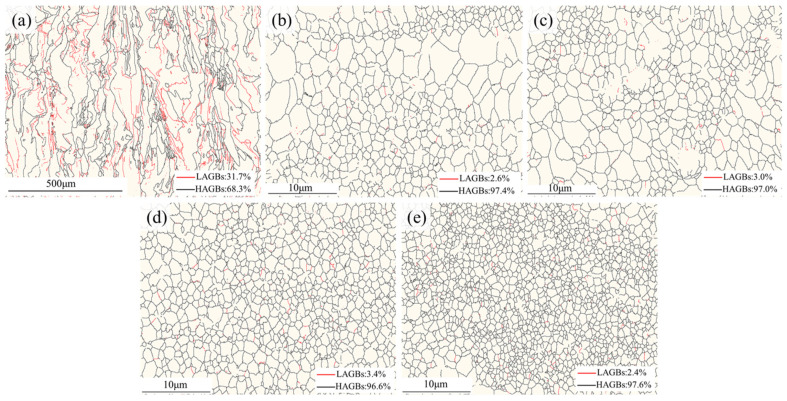
Grain boundary distribution maps on the vertical cross-sections of the samples with different initial TiH_2_ mass fractions: (**a**) 0 wt.%, (**b**) 1.0 wt.%, (**c**) 1.5 wt.%, (**d**) 3.0 wt.%, and (**e**) 4.5 wt.%.

Figure 17 illustrates the grain boundary distributions of the samples. In the unmodified 0 wt.% group, the proportion of low-angle grain boundaries (LAGBs, misorientation < 15°) was relatively high, reaching 31.7%. This is primarily attributed to the rapid thermal cycling experienced by each molten pool and the immense thermal contraction stresses generated by the rapid solidification inherent to the LPBF process. Such stresses are difficult to release effectively within the coarse columnar grains, leading to severe intragranular dislocation entanglement and consequently inducing the formation of numerous subgrain boundaries [45]. In contrast, upon modification (1.0–4.5 wt.%), the proportion of high-angle grain boundaries (HAGBs, misorientation > 15°) surged rapidly and stabilized between 96.6% and 97.6%. This demonstrates that the observed grain refinement was not merely the result of intragranular structural fragmentation or polygonization. Rather, the Al_3_Ti particles triggered massive, independent, and complete heterogeneous nucleation events, culminating in the formation of new grains characterized by high-angle grain boundaries.

**Figure 18:**
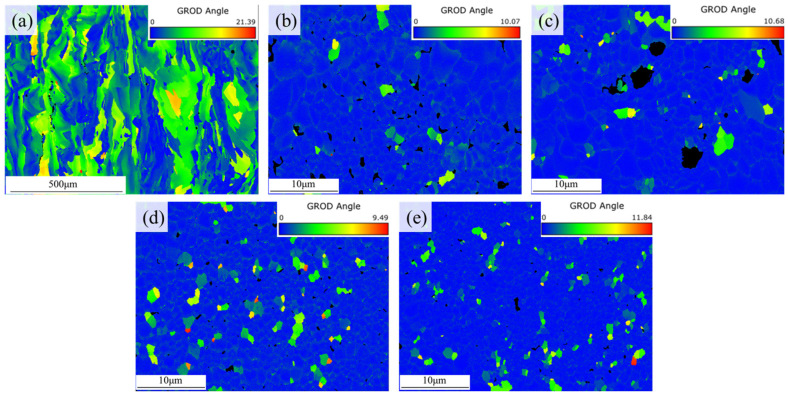
GROD maps on the vertical cross-sections of the samples with different initial TiH_2_ mass fractions: (**a**) 0 wt.%, (**b**) 1.0 wt.%, (**c**) 1.5 wt.%, (**d**) 3.0 wt.%, and (**e**) 4.5 wt.%.

Figure 18 illustrates the Grain Reference Orientation Deviation (GROD) of the samples, which is commonly utilized to visually reflect the accumulation of geometrically necessary dislocations (GNDs) and the concentration of local residual stresses within the grains. In the 0 wt.% group, the coarse and interlocked columnar grains failed to effectively accommodate the severe thermal contraction during the late stages of solidification, leading to the generation of extremely high local residual stresses, with the maximum GROD value reaching 21.39°. According to the solidification cracking criterion proposed by Kou [46], such a coarse columnar grain network is not only incapable of accommodating tensile stresses through inherent grain deformation during the terminal solidification phase, but its narrow and enclosed interdendritic channels also severely impede the timely feeding of the residual solute-rich liquid phase. Consequently, strains are highly concentrated at the long, straight high-angle grain boundaries, directly inducing the tearing of grain boundary liquid films and the initiation of macroscopic solidification cracks. After modification, the fine, randomly oriented equiaxed grain network induced by the abundant Al_3_Ti particles could effectively enhance the strain accommodation capacity of the matrix through various mechanisms, including grain boundary sliding, slight grain rotation, and microscopic plastic deformation. Therefore, the maximum GROD value of the 3.0 wt.% group decreased significantly to 9.49°, exhibiting a more uniform strain distribution and a favorable crack-resistant metallurgical state. However, in the 4.5 wt.% group, the extensive presence of hydrogen-induced gas pores weakened the structural continuity. Coupled with the local defect sources formed by the coarsening and agglomeration of Al_3_Ti particles, the maximum GROD value rebounded to 11.84°, indicating that new local stress concentrations were triggered at the edges of the micropores and coarse phases.

### 3.3. Mechanical Properties and Strengthening Mechanisms

#### 3.3.1. Compressive Deformation Behavior and Grain Refinement Strengthening Effect

**Figure 19:**
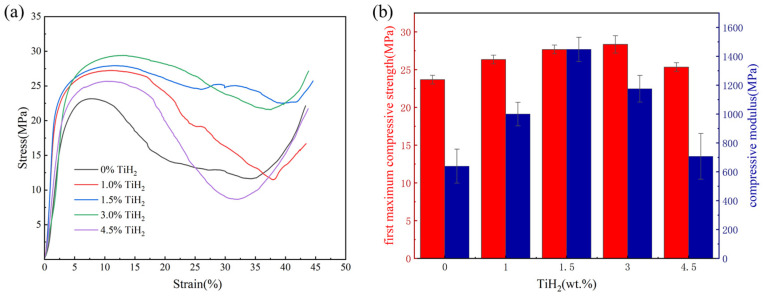
Compressive mechanical properties of the Diamond TPMS structures with different initial TiH_2_ mass fractions: (**a**) Quasi-static compressive stress–strain curves; (**b**) Comparison of the first maximum compressive strength and compressive modulus.

The quasi-static compressive stress–strain curves obtained using an AG-IC universal testing machine are presented in Figure 19a. The Diamond TPMS structures of all groups exhibited a typical mechanical response for porous materials, consisting of a linear elastic deformation region, a long stress plateau region, and a final densification region. It should be pointed out that, constrained by the printed build volume in this study, the compression specimens contained a relatively small number of unit cells (2 × 2 × 2). Boundary effects might cause the measured absolute values of the elastic modulus and plateau stress to exhibit a certain deviation from those of macroscopic bulk materials. However, since all comparative groups adopted the same external dimensions and lattice configurations, this relative mechanical evolution trend can still accurately reflect the contribution of microstructural evolution to the strength and toughness of the structure. Figure 19b indicates that the compressive elastic modulus and peak plateau stress of the porous structures decouple in their evolution trends with increasing TiH_2_ concentration, reaching their maximum values at 1.5 wt.% and 3.0 wt.% TiH_2_ additions, respectively.

As a typical porous material, the macroscopic elastic modulus of the TPMS structure primarily depends on the effective solid cross-sectional area and macroscopic geometric continuity of its structural skeleton. Figure 19b demonstrates that at the 1.5 wt.% addition, the compressive elastic modulus of the sample reached 1400 MPa, the maximum among all groups. This is attributed to the fact that at this concentration, the grain refinement effect induced by TiH_2_ was sufficient to effectively heal the fatal macroscopic solidification cracks present in the unmodified alloy (the 0 wt.% group suffered from severe internal cracking that severed its load-bearing cross-section, resulting in a compressive elastic modulus of only 600 MPa, the lowest among all groups), thereby restoring the geometric continuity of the structural skeleton. Meanwhile, compared to the groups with higher addition concentrations, the hydrogen-induced porosity generated by the dehydrogenation reaction in the 1.5 wt.% group was better controlled, yielding fewer microscopic pores. Therefore, the high internal densification of the structural skeleton in this group endowed the structure with excellent resistance to elastic deformation.

Although the elastic modulus of the 3.0 wt.% group (approximately 1150 MPa) was lower than that of the 1.5 wt.% group due to the increased number of internal pores, it exhibited a higher compressive peak plateau stress (approximately 28.5 MPa) after entering the plastic yield stage. This significant enhancement in post-yield strength indicates that the compressive strength of the sample is no longer governed by minor elastic porosity but is dominated by evident microscopic metallurgical strengthening mechanisms within the matrix. As shown in Figure 15, the average grain size in the 3.0 wt.% group was refined to approximately 1.0 µm. The substantially increased high-angle grain boundary (HAGB) network constitutes an effective physical barrier impeding dislocation slip. Abundant grain boundaries can effectively arrest dislocation movement and promote dislocation pile-up at the boundaries, thereby dramatically elevating the yield strength of the material. This strengthening increment follows the classic Hall–Petch relationship [31]:

(4) $$∆\sigma_{Hall-petch}\;=\;Kd^{-1/2}$$

where K is the Hall–Petch constant, and *d* is the average grain size of the matrix.

Furthermore, the EBSD phase maps presented in Figure 13 demonstrate that a high density of fine Al_3_Ti precipitates formed in the 3.0 wt.% group, uniformly dispersed throughout the aluminum matrix. Compared to the unmodified 6063 aluminum alloy, these precipitate particles, which possess high hardness and excellent thermal stability, can effectively impede dislocation movement through the Orowan bypassing mechanism during deformation under load, thereby enhancing the yield strength of the material [47].

As shown in Figure 8b, although the surface roughness increases with the increase in TiH_2_ mass fraction, the surface roughness of the samples does not change much when a low mass fraction is added (such as 1.0 wt.%, 1.5 wt.%), with Sa only increasing from 28 μm to approximately 34.6 μm. Therefore, the compressive mechanical properties of the samples are mainly dominated by the fine-grain strengthening caused by Al_3_Ti. However, when the TiH_2_ mass fraction increases to 4.5 wt.%, the surface roughness increases significantly, with Sa reaching 66 μm. This severe surface undulation forms a serious “geometric notch” on the thin-walled pillars. Under loading, the extremely high local stress concentration caused by the notch leads to premature local buckling and fracture of the pillars. This “notch effect” works in concert with the internal surge of microvoid defects, completely offsetting the strengthening benefits brought by grain refinement, ultimately resulting in a significant decline in the macroscopic compressive mechanical properties of this group of samples.

#### 3.3.2. Energy Absorption Characteristics

Energy absorption capacity is a core indicator for evaluating the application potential of TPMS porous structures in the fields of impact resistance, crash protection, and mechanical buffering [48]. Based on the uniaxial compressive stress–strain curves, the energy absorption performance of TPMS porous structures is calculated using the following equation [49]:

(5) $$W_{V}\;=\;\int_{0}^{\varepsilon }\sigma (\varepsilon )d\epsilon$$

where *σ* denotes the stress, *ε* represents the strain, and *W_V_* is the energy absorbed per unit volume by the porous structure.

**Figure 20:**
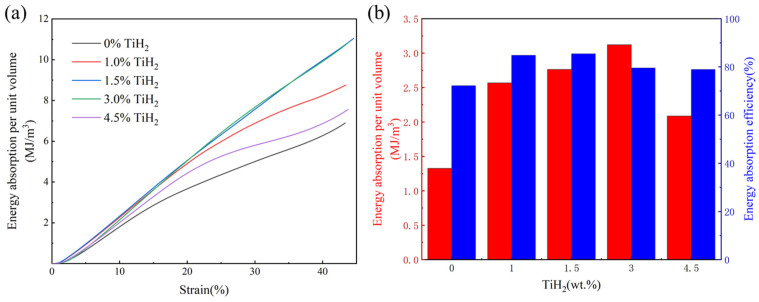
Compressive energy absorption characteristics of Diamond TPMS structures with different initial TiH_2_ mass fractions: (**a**) Cumulative energy absorption per unit volume versus strain curves; (**b**) Comparison of total energy absorption per unit volume and energy absorption efficiency.

Figure 20a illustrates the energy absorption per unit volume for the five groups with different TiH_2_ additions under various compressive strains. During the elastic deformation stage, the energy absorption values for all samples increased linearly and were approximately equivalent. Upon entering the stress plateau and subsequent failure stages, the energy absorption values followed the order: 3.0 wt.% ≈ 1.5 wt.% > 1.0 wt.% > 4.5 wt.% > 0 wt.%. This ranking is primarily attributed to the superior peak plateau stresses and elastic moduli of the 3.0 wt.% and 1.5 wt.% groups. Furthermore, the energy absorption curves for these two groups are smooth and devoid of significant fluctuations, indicating excellent ductility and stable energy absorption performance [50].

Figure 20b presents the energy absorption per unit volume during compression up to the upper strain limit (the strain corresponding to the peak plateau stress). The 3.0 wt.% group exhibited a high energy absorption value, confirming a favorable combination of ultimate load-bearing capacity and total energy absorption. Given that all experimental groups utilized the same Diamond macro-topology, the fundamental reason for the 3.0 wt.% group demonstrating the optimal energy absorption characteristics lies in the microstructural optimization triggered by the appropriate TiH_2_ addition, which effectively leveraged the inherent energy absorption potential of the Diamond lattice structure [50]. Research indicates that the energy absorption performance of porous lattice structures under compression depends not only on the cell configuration but also significantly on the strength-toughness and defect tolerance of the matrix material itself [51]. In the unmodified (0 wt.%) sample, severe macroscopic solidification cracks within the matrix acted as prominent stress concentration sites, leading to localized fracture or premature collapse of the structural skeleton during the early loading stages, thereby failing to exploit the mechanical advantages of the Diamond structure.

In contrast, for the 3.0 wt.% group, appropriate TiH_2_ doping not only effectively healed the primary cracks and ensured the physical continuity of the skeleton, but also achieved significant grain refinement and in situ strengthening of the matrix via high-density, dispersedly precipitated Al_3_Ti particles [52]. This uniform and robust microstructure enhanced the plastic flow stress and deformation coordination capacity of the aluminum alloy matrix. Consequently, the Diamond lattice struts could continuously sustain and transmit higher loads during large bending-shear deformations without the easy initiation of microcracks, thereby exhibiting superior energy absorption performance. On the other hand, the 1.5 wt.% group exhibited a high energy absorption efficiency of up to 85.5%, which is closely related to its superior structural continuity, relatively lower internal porosity, and maximum elastic modulus.

However, when the TiH_2_ concentration further increased to 4.5 wt.%, the surge of nearly 50,000 micropores severely compromised the continuity of the solid structure. Additionally, the coarse and agglomerated Al_3_Ti particles revealed in Figure 13d easily triggered stress concentrations under loading, acting as sites for microcrack initiation and propagation. The cumulative negative effects of these defects outweighed the benefits of grain refinement, leading to a significant decline in the elastic modulus, peak stress, and energy absorption values for this group.

### 3.4. Thermal Conductivity and Heat Dissipation Performance

In solid-state physics, heat transport within metallic materials primarily depends on the migration of free electrons and the propagation of lattice vibrations (phonons) [53]. According to Matthiessen’s rule, the total thermal resistance of a material is the superposition of various scattering mechanisms. The intrusion of foreign solute atoms (such as Ti atoms dissolved into the Al lattice) disrupts the original periodic arrangement of the lattice, causing significant lattice distortion. These distortion sites act as efficient scattering centers for both free electrons and phonons, thereby significantly reducing the intrinsic thermal conductivity of the material [54].

**Figure 21:**
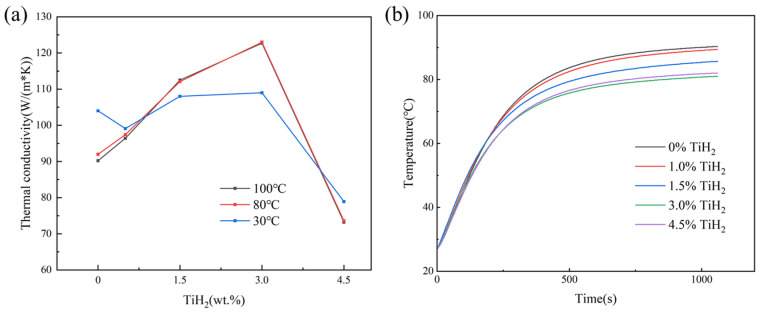
Characterization of thermal properties for samples with different initial TiH_2_ additions: (**a**) intrinsic thermal conductivity of the dense matrix measured at 30 °C, 80 °C, and 100 °C; (**b**) real-time temperature rise profiles of porous structure samples during heating at a constant power output of 4.0 W.

Figure 21a illustrates the thermal conductivity of thin-plate samples with different TiH_2_ additions at 30 °C, 80 °C, and 100 °C (it should be noted that this thermal conductivity reflects the intrinsic property of the modified dense matrix material). For the 1.0 wt.% group, due to the extremely high cooling rates inherent to LPBF, a portion of the Ti atoms that failed to participate in the nucleation reaction was forcibly trapped within the matrix in a solid solution state. This increased the scattering cross-section for electrons and phonons, resulting in a lower thermal conductivity for the 1.0 wt.% group at 30 °C compared to the unmodified 0 wt.% group. However, since defect scattering caused by solid solutions is relatively insensitive to temperature changes, its thermal diffusivity decreases at a slower rate with rising temperature than that of the pure Al group. Consequently, the thermal conductivity of the 1.0 wt.% group eventually surpassed that of the 0 wt.% group in the high-temperature ranges of 80 °C and 100 °C.

Furthermore, as the TiH_2_ addition increased to 3.0 wt.%, the thermal conductivity improved steadily and remained at a high level in the 80 °C and 100 °C ranges, with the 3.0 wt.% group reaching 123 W/(m·K) at 100 °C. Two primary factors contribute to this thermal performance enhancement: First, the appropriate addition of 3.0 wt.% TiH_2_ effectively healed the penetrating microcracks present in the 0 wt.% sample, re-establishing the macroscopic continuous conduction paths for electrons and phonons through the solid matrix [55]. Second, Figure 13 demonstrates that the density of Al_3_Ti phases increased with higher TiH_2_ concentrations. The elevated Ti concentration provided a greater thermodynamic driving force, prompting the majority of Ti elements to transition from the lattice solid solution state into Al_3_Ti precipitates. This reduction in solid solution content weakened the intrinsic scattering effect of lattice distortion on electrons and phonons, which is beneficial for the thermal conductivity of the samples [18].

Figure 21b displays the temperature rise curves obtained from simulated steady-state temperature tests of the porous structures at a constant power output of 4.0 W. The results show that the unmodified AA6063 TPMS reached an external steady-state temperature of 90.8 °C due to cracks obstructing internal heat conduction. In contrast, with the 3.0 wt.% TiH_2_ modification, the steady-state temperature dropped to 80.5 °C. This improvement is mainly attributed to the favorable physical coupling between the purified matrix with higher thermal conductivity and the complex, tortuous internal flow channels of the Diamond TPMS. During the convective heat transfer process, the fluid is forced into the non-straight channels of the Diamond structure, inducing local secondary flows and vortices. This enhances the agitation of the fluid near the walls, thereby intensifying heat transfer [56]. Additionally, as shown in Figure 8b, the 3.0 wt.% group exhibited relatively high surface roughness. Related studies suggest that the inherent roughness of additive manufacturing surfaces can increase the actual effective heat exchange area, thus improving the surface convective heat transfer coefficient [57]. When the addition reached 4.5 wt.%, the surge of high-density micro-scale hydrogen pores re-introduced internal thermal blockage effects, causing the thermal conductivity at 100 °C to fall back to approximately 73.2 W/(m·K) and leading to a rebound in the external steady-state temperature to 82.1 °C.

A comprehensive assessment of mechanical and thermal properties indicates that although the 1.5 wt.% group has advantages in elastic modulus and energy absorption efficiency, the 3.0 wt.% addition level is determined to be the optimal modification level based on multifunctional standards. Although its macroscopic stiffness and energy absorption efficiency are slightly lower compared to the 1.5 wt.% group, it achieves the highest plateau stress and thermal conductivity (28.5 MPa and 123 W/(m·K), respectively). Additionally, by achieving a fully healed matrix microstructure, the 3.0 wt.% group ensures structural integrity while enhancing mechanical and thermal properties, making it the preferred candidate material for the integrated load-bearing and heat dissipation TPMS architecture.

The main properties of the samples of each component are shown in Table 4.

## 4. Conclusions

This study systematically investigated the microstructural evolution and synergistic thermo-mechanical performance of AA6063 TPMS Diamond porous structures modified with varying TiH_2_ contents (0, 1.0, 1.5, 3.0, and 4.5 wt.%), fabricated via LPBF at a volumetric energy density (VED) of 60.85 J/mm^3^.

The primary conclusions are as follows:

1. CET and crystallographic mechanisms: Phase maps corroborated the in situ formation of Al_3_Ti particles. At appropriate doping levels, these particles served as effective heterogeneous nucleation sites, promoting grain refinement from 30.5 µm to approximately 1.0 µm and facilitating a significant columnar-to-equiaxed transition (CET). GROD analysis indicated that this fine equiaxed grain network mitigated residual thermal strain concentration (local misorientation reduced from 21.39° to 9.49°), thereby inhibiting the initiation of solidification cracks.
2. Evolution of pore defects and physical trade-offs: Metallographic and CT quantitative statistics on the XOY plane revealed that internal microporosity remained at a low level within the 1.0–3.0 wt.% range. However, at 4.5 wt.%, the excessive dehydrogenation reaction caused the number of small hydrogen-induced pores (<20 µm) to surge to nearly 50,000. This was accompanied by the coarsening and agglomeration of Al_3_Ti particles and an increase in macroscopic surface roughness, with the Sa value rising to 56 µm.
3. Decoupling of thermo-mechanical properties and the optimal solution: The 1.5 wt.% group exhibited a high compressive elastic modulus and energy absorption efficiency due to minimal pore defects. Comprehensive evaluation identified 3.0 wt.% as the optimal addition level. Mechanically, it achieved synergistic Hall–Petch and Orowan strengthening via uniformly dispersed Al_3_Ti, yielding a high peak plateau stress (28.5 MPa) and cumulative energy absorption (3.1 MJ/m^3^). Thermally, the dual effects of crack healing (eliminating thermal resistance) and matrix purification (via extensive Al_3_Ti precipitation) enhanced the thermal conductivity to 123 W/(m·K) at 100 °C. In steady-state heat dissipation tests with a 4.0 W heat source, this group achieved a lower operating temperature (80.5 °C), supported by the enhanced convective heat transfer resulting from the increased surface roughness.

## Figures and Tables

**Table 1:** Chemical composition of the AA6063 alloy powder.

Element	Al	Mg	Si	Fe	O
Content (wt.%)	Bal.	0.821	0.237	0.116	0.033

**Table 2:** Model parameters of the TPMS lattice structure.

Model Category	Model Design Volume Fraction (%)	The Actual Volume of the Model (mm^3^)	The Actual Volume Fraction of the Model (%)	Surface Area of the Model (mm^2^)
Diamond	30	1007.661	29.837	3829.800

**Table 3:** Main processing parameters of the LPBF process.

Laser Power (W)	Scanning Velocity (mm/s)	Layer Thickness (μm)	Hatch Spacing (μm)	Hatch Angle (°)
230	1400	30	90	67

**Table 4:** Main properties of samples with different TiH_2_ mass fractions.

Mass Fraction of TiH_2_ (wt.%)	Average Grain Size (μm)	Peak Plateau Stress (MPa)	Energy Absorption Efficiency (%)	Thermal Conductivity (at 100 °C) (W/(m·K)	Steady-State Temperature (°C)
0	30.46	23.7	72.2%	90.2	90.8
1.0	1.82	26.3	84.8%	96.4	89.4
1.5	1.25	27.6	85.5%	112.5	85.7
3.0	1	28.5	79.6%	123	80.5
4.5	0.75	25.3	78.9%	73.2	82

## Data Availability

The original contributions presented in this study are included in the article. Further inquiries can be directed to the corresponding author.
